# Tri-layer microbiology for LMIC Hospitals: linking syndromic panels with reflex culture and targeted sequencing for real world care - a narrative review

**DOI:** 10.1016/j.bjid.2026.105808

**Published:** 2026-04-08

**Authors:** Tokeshwar Kumar Sahu, Jaishriram Rathored, Praful Patil

**Affiliations:** a Datta Meghe Institute of Higher Education & Research, Jawaharlal Nehru Medical College, Department of Microbiology, Sawangi Meghe, Wardha, India; b Central Research Laboratory and Molecular Diagnostics, Jawaharlal Nehru Medical College, Datta Meghe Institute of Higher Education & Research, Sawangi Meghe, Wardha, India; c School of Allied Health Sciences, Datta Meghe Institute of Higher Education & Research, Sawangi Meghe, Wardha, India

**Keywords:** Antimicrobial stewardship, Rapid diagnostic tests, Reflex culture, Metagenomic next-generation sequencing, Diagnostic stewardship

## Abstract

Rapid, syndromic molecular panels and high throughput sequencing have transformed the diagnostic landscape for sepsis, respiratory, gastrointestinal, and central nervous system infections, but their value in routine practice depends on how they are integrated with conventional microbiology and antimicrobial stewardship. This review synthesises recent high-quality evidence to propose a pragmatic three-tier hybrid framework. Tier 1 comprises syndrome specific rapid panels that provide organism and selected resistance markers within hours, primarily to accelerate early escalation or de-escalation rather than to replace culture. Tier 2 positions reflex culture and targeted adjunct tests as the non-negotiable specificity anchor, confirming molecular hits, distinguishing infection from colonisation or contamination, generating phenotypic susceptibility data and supplying isolates for infection prevention and public health surveillance. Tier 3 reserves targeted or metagenomic sequencing for a small, clinically critical subset of high suspicion, panel negative and culture negative cases, where additional breadth can realistically change management. Across sepsis/BSI, pneumonia, gastrointestinal infection and CNS disease, available data indicate that clinical benefit is driven less by any individual technology and more by disciplined implementation: clear indications, explicit reflex rules, close linkage to antimicrobial stewardship and systematic audit of key performance indicators such as time-to-targeted therapy, spectrum of antimicrobial use and cost per additional actionable diagnosis. The proposed tiered, syndrome wise algorithms provide a transferable conceptual scaffold that can be adapted to local resources, allowing laboratories in both high and low resource settings to introduce advanced diagnostics without abandoning culture-based anchors or stewardship accountability.

## Introduction

Microbiology laboratories across Low and Middle Income Countries (LMICs) operate under chronic budget ceilings, intermittent power, limited maintenance coverage, and constrained informatics pipelines that can delay clinician facing results and blunt clinical impact.^1^ Real world ICU experiences from tertiary centers further show that platform uptime, sample flow, and trained personnel determine whether advanced assays translate into actionable decisions, reinforcing that implementation architecture not technology alone drives outcomes.^2^

No single modality adequately addresses these constraints.

Culture remains indispensable for confirmation and phenotypic antimicrobial susceptibility testing, but slow time to result and post antibiotic false negativity limit its utility for early therapy optimization.^2^

Syndromic multiplex panels compress time to result from days to hours and cover common targets, yet they are bound to predefined menus multicenter evaluations of blood culture ID panels-report excellent on panel performance while highlighting clinically relevant off panel organisms that necessitate parallel/reflex culture.^3^

In respiratory care, panels can surface nucleic acids from colonizers or nonviable organisms: without semi quantitative interpretation and reflex steps, overtreatment becomes a real risk.^4^

Sequencing (targeted or metagenomic) extends breadth and helps in culture negative or atypical infections, but requires bioinformatics capability, contamination safeguards, and budget headroom making it best positioned as an escalation rather than universal front door in LMIC labs.^5^

These complementary strengths and weaknesses motivate a tri-layer diagnostic strategy: i) A rapid syndromic panel to rule in likely etiologies within hours. ii) Reflex culture as the specificity/AST anchor for confirmation and infection control actions and (iii) Targeted sequencing for unresolved, high suspicion scenarios.^6^

In sepsis pathways, embedding a blood culture ID panel within stewardship workflows has been associated with markedly shorter time to targeted therapy demonstrating how the first layer accelerates decisions while subsequent layers add depth and precision.^7^

ICU experience from Indian tertiary settings similarly shows that panel calls are most valuable when explicitly coupled to escalation de-escalation rules and confirmation by culture, underscoring that complement, not replace, culture.^2^

The clinical endpoints that matter in LMIC hospitals are concrete and trackable: diagnostic yield**,** time to effective therapy, days of broad-spectrum exposure, isolation days**,** length of stay, and readmissions.^8^

In emergency and ICU respiratory cohorts, panel guided management has reduced time to appropriate therapy and antibiotic exposure when tied to stewardship oversight, highlighting that rapid results must be embedded in governance to shift outcomes.^9^^,^^10^

ED focused evaluations likewise show pneumonia panels can accelerate actionable decisions but require reflex culture and clinical context to arbitrate colonization versus disease.^11^

In gastrointestinal pathways, selective reflex culture after panel positives improves specificity and infection control value while avoiding blanket culture of all specimens, with modelled reductions in technologist time and total costs critical signals for budget limited labs.^12^^,^^13^

Conversely, pediatric LMIC analyses caution that indiscriminate panel use without indication criteria and audit loops can strain budgets, emphasizing the need for disciplined test utilization and KPI dashboards.^14^

This review targets microbiologists, infectious disease clinicians, and administrators in tertiary and district LMIC hospitals**,** with emphasis on ED/ICU where decisions are time critical and stewardship consequences largest.

Contribution We synthesize a) Modality specific strengths and pitfalls, b) Reflex rules that operationalize a tri-layer algorithm, and c) A pragmatic implementation playbook (people, process, platform) including LIS prompts and audit cadence.

We also propose a concise set of KPI hooks time to effective therapy, DOT reduction, isolation days saved, and LOS/readmissions so programs can quantify impact and iterate locally under LMIC constraints.^12^^,^^15^

## Syndromic panels in routine practice: strengths vs. traps

Syndromic multiplex assays (respiratory, gastrointestinal, blood culture ID, and CSF/meningitis panels) compress the diagnostic timeline from days to hours, enabling organism level signals during the first decision window of care.^7^^,^^16^ In emergency and ICU cohorts with suspected respiratory infection, panel enabled stewardship has been associated with faster time to appropriate therapy and reduced unnecessary broad spectrum exposure when results are embedded in governance pathways rather than used in isolation.^9^

Technically, these platforms contribute three pragmatic advantages. Speed: closed, cartridge style workflows deliver same shift outputs for high acuity patients.^7^ Breadth: curated, syndrome matched target menus often increase early etiologic yield relative to single target methods.^17^ Operational simplicity: standardized, low hands on steps are valuable where staffing is thin and shift variability is high.^17^ Semi quantitative binning on some respiratory panels further supports clinical correlation by down weighting low level colonizers when pre-test probability is modest.

These gains, however, come with predictable traps if panels are applied uncritically. Target menu limitation: clinically important off panel organisms will be missed even when the assay performs excellently on panelled targets necessitating parallel or reflex culture.^16^ Colonization vs. infection: nucleic acids from airway commensals or nonviable organisms may inflate positive calls: without semi quantitative interpretation and clinical context, overtreatment is likely.^18^ Co detections multiple hits are common in respiratory and GI pathways and can distort perceived complexity unless adjudicated with host factors, imaging, and biomarkers.^17^ Resistance inference limits**:** although some panels-report key resistance genes, stewardship decisions generally still require phenotypic susceptibility testing reinforcing the need to complement (not replace) the panel layer with culture.^16^

Accordingly, interpretation anchors should be explicit in routine use. i) Pre-test probability (syndrome severity, host factors, and imaging). ii) Sample quality and site, and iii) Infection control context (will the result change isolation or source control In respiratory and gastrointestinal workflows, structured reflex culture after panel positives restores specificity, enables phenotypic AST, and supports outbreak/infection control actions two pragmatic frameworks describe selective (not universal) culture triggers.^12^^,^^15^ Programs in resource limited settings should also codify utilization rules (indications, repeat testing boundaries, and actions on low bin results) and maintain an audit cadence, because indiscriminate testing can strain budgets without improving outcomes.^13^ Bottom line: panels are powerful rule in accelerators, but they deliver safe, stewardship aligned value only inside a tri-layer workflow where reflex culture and, when necessary, targeted sequencing provides specificity and resistance resolution.^7^^,^^12^

## Reflex culture: the specificity anchor

Reflex culture remains the cornerstone for adjudicating true infection versus colonization and for delivering phenotypic Antimicrobial Susceptibility Testing (AST) capabilities that no rapid panel can fully replace.^16^ In routine workflows, panel positive results should trigger targeted culture of the same specimen (or an anatomically appropriate companion specimen) to confirm organism identity, quantify growth, and generate AST within reporting windows that support de-escalation rather than merely documenting resistance late in the course.^12^^,^^15^ This panel culture handshake restores clinical specificity when multiplex assays detect nucleic acids from nonviable or colonizing organisms, a known source of overtreatment in respiratory and gastrointestinal pathways.^19^

Operationally, reflex culture adds three elements that convert an early molecular signal into a safe, stewardship aligned decision. First, organism confirmation with burden context: culture growth (including semi quantitative reporting or colony counts where relevant) helps discriminate pathogen from bystander, particularly when panels-report low bin or borderline detections.^15^^,^^20^ Second, phenotypic AST: while select panels carry resistance gene calls, actionable narrowing typically hinges on MIC based or disk diffusion data reflex culture supplies this within a defined window so that clinicians can de-escalate before day 3‒4.^12^ Third, infection control signal quality: culture enables strain level or phenotype linked alerts (e.g., ESBL, MRSA, carbapenemase phenotypes) that drive isolation and cohorting decisions, often more reliably than gene surrogates alone.^21^

Reflex rules should be disease and target specific rather than universal. For bloodstream infection pathways, any panel detected pathogen or resistance determinant that would change therapy or isolation merits immediate subculture from the blood culture bottle, plus directed plates for fastidious organisms contamination controls (e.g., single bottle coagulase negative staphylococci) should be codified to avoid unnecessary workups.^22^ In respiratory syndromes, laboratories should culture panel positive lower respiratory specimens when semi quant bins are moderate/high or when clinical severity is high for low bin detections in low pre-test settings, selective culture paired with clinical correlation curbs overtreatment and unnecessary AST.^23^ For gastrointestinal panels, selective reflex culture of bacterial positives (e.g., Salmonella/Shigella/Campylobacter) enables susceptibility reporting and public health flags without reverting to blanket culture of all stools this approach has demonstrated reductions in technologist time and overall costs when implemented with rules.^12^

Turnaround Time (TAT) discipline is central. Molecular positives should be released rapidly with an embedded comment that reflex culture is in progress and that definitive AST will follow by a specified deadline, aligning clinicians’ expectations with laboratory kinetics.^15^ Many programs have standardized “rapid phone out” for critical targets (e.g., S. aureus in blood, N. meningitidis in CSF) simultaneous with setting up reflex culture, ensuring early source control while preserving the pathway to phenotypic confirmation.^24^ In LMIC settings, LIS prompts and rule based order sets reduce missed reflexes during busy shifts and mitigate variability across staff with differing experience levels.^15^

From a cost utility perspective, reflex culture is not a luxury it is the specificity and stewardship engine that protects panel investments. Modelling work shows that selective reflex strategies (rather than universal culture) can lower total laboratory costs and hands on time while preserving clinical utility an important balance where budgets are fixed and technologist capacity is limited.^12^^,^^13^ The practical message for LMIC hospitals is to deploy reflex culture with disciplined triggers, clear contamination rules, and defined reporting windows so that phenotypic data land early enough to change management.^25^

## Targeted sequencing as a reflex step

### Position on the diagnostic ladder

In LMIC hospital workflows, sequencing is most effective as a third tier escalation after a rapid syndromic panel and reflex culture, specifically when clinical suspicion remains high yet routine methods are non-diagnostic e.g., culture negative sepsis or endocarditis, panel negative meningitis/encephalitis, and polymicrobial lower respiratory infections.^26^^,^^27^

Feasibility across LMIC tiers (district vs. tertiary): In practice, this third tier will not look the same across LMIC hospitals. Most district laboratories will not be able to run sequencing on-site because the “hidden” requirements (stable power, segregated work areas, quality controls, contamination monitoring, trained bioinformatics, and governance for interpretation/reporting) are as important as the sequencer itself. A more realistic model is hub-and-spoke delivery: district sites follow Tier 1‒2 (panel + reflex culture) and refer pre-defined reflex indications to a regional/tertiary reference hub that can maintain validated pipelines and provide interpretive support. This framing keeps sequencing as an escalation option without assuming that every hospital must build and sustain a full sequencing stack.

### Why reflex (and not front door)

Rapid panels shorten time to organism ID and guide early stewardship, but they inevitably leave residual risk (off menu taxa, prior antibiotic suppression, fastidious organisms) that sequencing can resolve without a priori targets.^16^

### Modality choice tNGS versus mngs

Targeted amplicon/capture sequencing (tNGS**)** concentrates reads on curated loci, delivering lower cost, simpler analytics, and predictable turnaround well aligned to LMIC constraints when reflex indications are clearly defined.^26^ Metagenomic Sequencing (mNGS**)** provides hypothesis free breadth and adds yield in culture negative CNS/sepsis and complex lower respiratory specimens, but demands stricter contamination control and bioinformatics.^27^ A staged pathway tNGS first, mNGS for unresolved or high severity cases balances feasibility with breadth.^27^

### Pre analytical safeguards and interpretation

Low biomass matrices (e.g., CSF, plasma) require extraction blanks, no template controls, unidirectional workflow, and index hygiene to minimise environmental DNA carry over results should be released only above pre specified abundance/coverage thresholds and interpreted against clinical context.^28^

### Turnaround and reporting

For urgent reflex indications, laboratories should target < 24‒48h sample to report using streamlined pipelines or referral lanes clinician facing reports must include organism call with confidence, normalised abundance/coverage, validated resistance loci (if applicable), and explicit limitations.^28^

### Clinical utility signals

In bloodstream infection pathways, the addition of an early panel tier (with culture) has already demonstrated substantial reductions in time to targeted therapy and favourable 30-day outcomes adding sequencing for the unresolved fraction leverages the same stewardship backbone rather than replacing it.^7^ In culture negative scenarios, plasma microbial cell free DNA sequencing has expanded etiological diagnosis across multiple syndromes and informed targeted therapy.^26^

### Actionable reflex rules

1) Culture negative sepsis/CNS at 48‒72 h with high suspicion: trigger sequencing if positive, pursue back culture for phenotype when feasible.^27^
2) Polymicrobial/conflicting panel signals (respiratory or BSI): use sequencing to adjudicate dominance, then confirm key targets by culture for AST.^16^
3) Atypical/fastidious suspicion (e.g., nocardiosis, mycoses, endocarditis): perform tNGS on high yield tissues/fluids: escalate to mNGS if tNGS is unrevealing and clinical severity persists.^27^^,^^28^
4) Severe LRTI with negative panel/culture (ICU): where sequencing is available on-site or via a referral hub, consider early BAL tNGS/mNGS to capture off-menu/fastidious agents, with strict contamination controls and cautious interpretation of low-level reads; where sequencing is not accessible, document the limitation and prioritise optimised conventional work-up (repeat high-quality sampling and targeted cultures/assays guided by local epidemiology).^16^

#### KPIs to audit

i) Incremental diagnostic yield in reflexed negatives (sequencing positive after panel/culture negative).^27^ ii) Time to effective therapy from sampling to first targeted change (benchmarking pre vs. post sequencing).^7^ iii) Broad spectrum DOT avoided and repeat LP/BAL rate post implementation.^28^

## Syndrome-wise hybrid algorithms

### Sepsis/BSI

#### Backbone: rapid panel + culture in parallel

A blood culture identification panel run on positive bottles provides organism ID (± select AMR genes) within 1‒2 h, enabling early escalation/de-escalation however, confirmation and phenotypic AST via reflex culture remain non-negotiable for definitive care and stewardship.^7^^,^^29^

#### Impact with stewardship

After implementation of BCID2 pathways, multicentre ICU programmes have reported large reductions in time to optimal therapy and favourable trends in 30-day outcomes, particularly when results are embedded in real time stewardship notes and escalation protocols.^7^

Where sequencing adds value If cultures remain sterile at 48‒72 h while shock or organ failure persists, plasma/whole blood tNGS or mNGS can detect fastidious or partially suppressed pathogens a positive call should trigger targeted therapy and, where feasible, back culture from an appropriate matrix for phenotypic confirmation.^26^^,^^27^

### Actionable reflex rules (Sepsis/BSI)

High-impact panel hits (*Staphylococcus aureus, Candida* spp.*, Enterococcus* spp. or major β-lactamase markers): immediate subculture + AST and add a standard LIS comment indicating expected AST-TAT to cue de-escalation.^7^

Probable contaminant on panel (e.g., skin commensal in a single bottle): obtain repeat cultures before committing to prolonged therapy.^7^

Panel/culture negative at 48‒72h with ongoing sepsis: initiate cfDNA/tNGS or mNGS: if positive, align therapy and pursue isolate recovery where practical.^26^

Discordant or polymicrobial signals: use sequencing to identify the dominant pathogen signature before broadening empiric cover unnecessarily, then confirm by selective subculture for AST.^30^

#### KPIs (Sepsis/BSI)

i) Median time to targeted therapy pre vs. post implementation.^7^ ii) Aetiologic yield across tiers (culture alone vs. panel + culture vs. panel + culture + sequencing).^16^ iii) 30-day mortality trend to ensure clinical benefit matches process gains.^7^

### Respiratory

#### Rationale and tiering

Lower respiratory specimens are often polymicrobial and partially pre-treated: a rapid semiquantitative pneumonia PCR panel (on BAL/sputum) front loads organism calls and key AMR markers within hours, but phenotypic AST on targeted subculture remains mandatory to confirm viability and guide definitive therapy. The BioFire PN/PNplus panels expanded organism detection and AMR marker recovery across multicentre cohorts, while semiquantitative bins correlate imperfectly with culture CFU hence the need for culture adjudication and stewardship oversight.^31^, ^32^, ^33^

#### What the panel changes today

In adult BAL cohorts, PN panel adoption increased pathogen detections versus standard culture and suggested potential antibiotic de-escalation or discontinuation in 48% of cases stewardship programmes can leverage these early negatives (e.g., *MRSA/Enterobacterales* markers absent) to narrow cover safely.^32^

#### Known interpretive pitfalls

Semi quantitative bin values do not perfectly separate colonisation from infection and panel positivity may exceed culture recovery especially after prior antibiotics laboratories should pair reports with clear interpretive comments and algorithms that trigger culture confirmation for dominance and AST.^32^^,^^33^

Where sequencing fits (reflex). For panel negative/culture negative severe LRTI (especially ICU) or discordant polymicrobial signals, reflex to tNGS/mNGS on BAL can adjudicate off menu or fastidious agents: however, pre analytical controls and conservative reporting thresholds are essential to avoid over calling low level background.^34^

Operational note (avoid over-promising in LMIC settings): Respiratory mNGS is technically demanding because BAL/sputum are complex, contamination-prone matrices and interpretation can drift toward “signal chasing” if governance is weak. For many LMIC programmes, the safest pathway is to restrict sequencing to a small set of ICU-grade indications and deliver it through a validated tertiary/reference service that can enforce QC and provide clinically contextual reporting. District sites should treat sequencing as a send-out escalation, not as a routine step, and continue to anchor decisions on culture/AST and stewardship rules.

### Actionable rules (respiratory)

1. High risk hits (e.g., *S. aureus*, ESBL/Carb genes) on panel → immediate selective subculture for AST: de-escalate when markers/targets are absent and clinical risk is low.^32^
2. Panel positive at low bins without concordant microscopy/clinical fit → withhold broadening, perform targeted culture and reassess at 24‒48 h.^33^
3. Panel/culture at 48‒72 h with persistent severity → reflex BAL tNGS/mNGS with strict contamination controls: interpret alongside host/inflammatory context.^34^

#### KPIs

i) Time to de-escalation post panel reporting. ii) Agreement of high bins (≥10^7 copies/mL) with culture dominance. iii) Incremental diagnostic yield of reflex mNGS in panel/culture negative ICU pneumonias.^32^^,^^33^

### Gastrointestinal

#### Front door, not free for all

Modern multiplex GI panels detect bacterial, viral, and parasitic agents with high analytical performance, but inappropriate ordering (no diarrhoea, laxative use, duplicate *C. difficile* testing, >72 h inpatient) dilutes clinical value an EMR embedded Clinical Decision Support (CDS) reduced overall GI-panel ordering by 39% and significantly curtailed laxative associated inappropriate tests.^35^

#### Why culture is still needed

During outbreaks and for reportable bacteria (e.g., *Shigella, Salmonella*, STEC), laboratories should reflex to stool culture from panel positive samples for isolate recovery (public health typing) and phenotypic AST an approach used prospectively in paediatric ED response to *Shigella.*^36^

#### Interpretation cautions

Some low prevalence targets can show false positives on CIDTs, e.g., Vibrio spp., reinforcing the need for reflex culture/orthogonal confirmation before definitive therapy or notification.^37^ Measured clinical impact. Across settings, GI panels have shown faster aetiologic assignment and shorter length of stay/time to discharge in positives, but stewardship must avoid unnecessary antibiotics for self-limited viral detections or colonising *Escherichia coli* pathotypes.^35^^,^^38^

#### Where sequencing fits (narrow, reflex)

For immunocompromised hosts with persistent severe diarrhoea and panel/stool culture results, consider targeted sequencing (e.g., for difficult protozoa/rare viruses) with prespecified thresholds and negative controls: report with explicit limitations to avoid over interpretation of trace reads.^34^

### Actionable rules (GI)

1. Order GI panel only if documented acute diarrhoea (≥ 3 loose stools/24 h), no recent laxatives, and within < 72 h of admission: otherwise require stewardship approval.^35^
2. Panel positive reportables → automatic reflex culture for confirmation, typing, and AST: hold final public health reporting until isolate confirmation.^36^
3. Multi pathogen detections with weak clinical correlation → favour supportive care and avoid antibiotics unless a treatable bacterial target is dominant or confirmed by culture.^35^

#### KPIs

i) Inappropriate order rate pre/post-CDS. ii) Reflex culture recovery among reportable detections. iii) Antibiotic initiation/de-escalation aligned to actionable bacterial targets.^35^^,^^36^

### Central nervous system (CNS)

#### Clinical position

In suspected CNS infection, a multiplex ME panel on CSF can return organism calls within 1‒2 h, but target level sensitivity varies therefore it must be complementary to CSF culture and syndrome directed adjunct tests, not a stand-alone replacement for isolate recovery and AST.^39^

#### What the ME meta-analysis shows

A 19-study diagnostic test-accuracy synthesis reported high overall specificity (97%‒99%) with variable sensitivity across individual bacterial/viral targets, reinforcing the need for orthogonal confirmation when results conflict with the clinical picture.^39^

#### Where sequencing adds value

When ME panel/culture are non-diagnostic and suspicion remains high particularly encephalitis or culture negative meningitis CSF metagenomic sequencing (mNGS) offers hypothesis free breadth and has shown sustained add-on yield and actionability in a 7-year, 4828-sample UCSF clinical series.^40^

#### Performance and clinical impact of mngs

Contemporary evaluations demonstrate positive percent agreement versus composite conventional methods and frequent management changes when organisms are detected supporting mNGS as a reflex rather than first-line test.^41^

#### Hard-to-detect pathogens (e.g., TBM)

For tuberculous meningitis, where smear/culture/Xpert may be insensitive, CSF nanopore sequencing has shown higher diagnostic sensitivity with high specificity in clinical cohorts, enabling earlier targeted treatment in ME-/culture negative cases.^42^

#### Low-biomass safeguards

Because CSF is low biomass, laboratories must run extraction blanks/NTCs, maintain unidirectional workflow and index hygiene, and apply predefined abundance/coverage thresholds before reporting: these controls are standard to avoid over calling environmental DNA.^28^

## Actionable hybrid algorithm (CNS)

### Step 1 ‒ front door testing (time-critical)

Perform ME panel + CSF culture at lumbar puncture treat early positives but avoid committing to prolonged therapy on equivocal/low prevalence targets without clinical concordance.^39^

### Step 2 ‒ orthogonal confirmation & adjuncts

• Bacterial positives: pursue targeted subculture for AST/typing.
• Viral positives with poor syndrome fit (e.g., HHV-6): confirm by specific PCR/repeat testing before long antivirals.
• TBM suspicion: run Xpert MTB/RIF ± culture: if negative yet suspicion remains high, keep nanopore sequencing as reflex.^42^

### Step 3 ‒ reflex sequencing triggers

  • ME/culture negative at 24‒48h with ongoing encephalitis/meningitis features → CSF mNGS: if positive, align therapy and attempt back culture for phenotype where feasible.^39^^,^^40^
  • Immunocompromised/atypical hosts (fungal, nocardial, parasitic, unusual viruses) → earlier mNGS consideration.^41^
  • High suspicion TBM with negative conventional tests → nanopore sequencing reflex.^42^

### Step 4 ‒ reporting discipline (mNGS/nanopore)

  • Provide organism call + confidence, normalised abundance/coverage, resistance loci only where clinically validated, and explicit limitations (DNA ≠ viability: host DNA burden).^28^

### Audit KPIs (service quality)

1. Aetiologic diagnosis rate in ME/culture negative encephalitis after reflex mNGS, benchmarked to historical cohorts.^40^
2. Time from LP to organism directed therapy (panel=hours sequencing ≤48 h with streamlined pipeline).^41^
3. Appropriate de-escalation after confirmation/clarification of ME findings, avoiding treatment for low value positives.^39^

## Stewardship & cost-utility signals

High-cost diagnostics in bloodstream infection, pneumonia, gastrointestinal disease and CNS infection only deliver net benefit when they are tightly coupled to antimicrobial stewardship (AMS) rather than used as stand-alone technologies. A 2024 network meta-analysis of 88 studies in bloodstream infections showed that Rapid Diagnostic Tests (RDTs) improved survival only when combined with an active AMS programme, with RDT + AMS outperforming both conventional blood culture alone and blood culture + AMS for mortality and time to optimal therapy.^43^ Evidence hierarchy and interpretation note: The most reproducible gains in the RDT literature come from implementations where the test is paired with an explicit stewardship “action loop” rather than deployed as a stand-alone technology. However, much of the impact evidence (including BCID2 and pneumonia panel rollouts) is quasi-experimental (pre–post and observational), and effect sizes can be inflated by co-interventions that often arrive at the same time (updated empiric guidelines, new order sets, audit-feedback cadence, staffing changes). For this reason, reported reductions in time to optimal therapy or broad-spectrum exposure should be read primarily as the impact of a bundled pathway redesign (“test + workflow + AMS response”), with expected attenuation in settings where stewardship bandwidth is limited.

In sepsis/BSI, multiple BCID2 implementation studies have demonstrated substantial reductions (often >24‒30 h) in time from empirical therapy to optimal, targeted therapy when BCID2 results automatically trigger AMS review, compared with MALDI-TOF or conventional methods alone.^44^^,^^45^ Cost effectiveness analyses suggest that cartridge and platform costs are offset by lower broad spectrum days of therapy, shorter ICU/hospital stay and fewer downstream investigations when AMS consistently acts on RDT outputs.^46^

For pneumonia, real world evaluations of the BioFire Pneumonia Panel in ICUs show that semi quantitative results integrated with stewardship review facilitate early de-escalation or discontinuation of unnecessary antibiotics in a meaningful proportion of patients, while avoiding unsafe narrowing in high risk hosts.^47^^,^^48^ Similar principles apply to gastrointestinal panels: clinical decision support tools embedded in the EMR have reduced overall GI-panel ordering and significantly lowered inappropriate tests (e.g., laxative associated, no documented diarrhoea)**,** highlighting that diagnostic stewardship is essential to justify panel cost and prevent overtreatment of colonising or self-limited pathogens.^35^^,^^49^

For metagenomic sequencing, recent cohorts and reviews across sepsis, respiratory and CNS infections show that mNGS increases diagnostic yield and prompts management changes in roughly 20%‒40% of tested patients, particularly when used selectively in culture negative, high suspicion cases.^41^^,^^50^^,^^51^ While some tertiary-centre series report shorter treatment duration and lower total episode costs compared with prolonged empirical management, these economic signals are highly context-dependent and may not translate directly to LMIC hospitals where costing structures, bed-day valuation and downstream capacity differ. LMIC programmes should therefore treat cost offsets as uncertain and prioritise local budget-impact evaluation before expansion beyond strict reflex indications.

Collectively, these data imply that the hybrid tiered algorithms proposed in this review are only sustainable if laboratories and AMS teams co-design: i) Strict indication criteria and order sets for panels and sequencing, ii) Automatic AMS notification for high impact results, and iii) Routine audit of time to targeted therapy, broad spectrum DOT and cost per additional actionable diagnosis. Minimum stewardship capacity (pragmatic baseline for LMIC sites): For tiered pathways to translate rapid results into safer antibiotic decisions, programmes should define a minimum operating set: i) A named AMS focal person or small rota accountable for acting on high-impact calls (sepsis/ICU), ii) A rapid communication channel for time-critical results (phone/secure message/LIS alert) with a clear escalation pathway, iii) Syndrome-linked empiric and de-escalation rules anchored to the local antibiogram, and iv) A simple daily review touchpoint (even once per working day) for sepsis/ICU RDT outputs. Where this action loop is absent, rapid panels can increase organism detection without improving outcomes and may unintentionally increase antibiotic exposure through ungoverned responses to positive signals. LMIC-facing cost-utility framing (pragmatic and measurable): Because many LMIC hospitals operate under hard budget constraints, cost-utility should be expressed not only as “cost-effectiveness” but also as budget impact and affordability under realistic volumes. A practical approach is to report i) Direct programme cost per test episode (cartridge/reagents, QC/controls, labour time, maintenance, and basic IT/LIS support), ii) Actionability rate (proportion of tests that trigger a stewardship or IPC action), and iii) Downstream offsets that are plausibly attributable to governed use (broad-spectrum DOT avoided, avoidable repeat diagnostics, isolation days optimised, and length-of-stay change where credible). Importantly, savings may manifest as released capacity rather than cash savings; therefore, reporting should explicitly distinguish “financial savings” from “capacity gains” and avoid assuming that reduced LOS automatically converts to budget return in all LMIC settings.

## Implementation playbook for LMIC labs

Translating hybrid diagnostic algorithms into routine practice is constrained first by laboratory level barriers. A recent JAC perspective on rapid ID/AST technologies highlights high capital and per test costs, limited reimbursement, need for 24/7 skilled staff, IT integration, and the complexity of redesigning workflows as major reasons why many laboratories cannot insource rapid systems despite clear clinical benefits.^52^ An ASM Laboratory Practices Subcommittee report further emphasises that diagnostic stewardship responsibilities are being added to laboratories already facing workforce shortages and fragile infrastructure, making sustained implementation difficult without institutional investment and protected time.^53^ Workforce and competency (task-shifting without losing governance): Because LMIC laboratories often cannot staff 24/7 specialist microbiology coverage, a tiered workforce model is more realistic than an “expert-only” model. Core tasks can be distributed with clear boundaries: technologists execute standardized workflows (panel run, reflex culture set-up, critical value phone-out templates); a supervising microbiologist validates complex/discordant outputs and maintains QC; and an AMS liaison (medicine/pharmacy) converts high-impact results into time-bound treatment actions using agreed de-escalation rules. For sites without an on-site ID physician, a small rota of trained internal medicine/ICU clinicians supported by pharmacy can function as the AMS action loop, provided there is a documented escalation pathway for complex cases. Competency should be operationalized as short, auditable modules (sample quality checks, semi-quant interpretation, contamination control, reflex triggers, and report wording), with quarterly refreshers and a “second-reader” rule for high-stakes results (sepsis/CSF, IPC organisms, sequencing reports).

In Low and Middle Income Countries (LMICs), these barriers are multiplied by limited access to basic microbiology, unreliable power and cold chain, and the near absence of formal stewardship programmes in many hospitals. Narrative reviews and qualitative work from LMIC settings describe under resourced laboratories, fragmented supply chains and weak governance as key obstacles to embedding microbiology driven stewardship, even before high-cost molecular platforms are considered. Under these conditions, the immediate priority is often to secure reliable culture, AST and basic quality systems, and to link them to pragmatic AMS rather than to deploy broad panels or mNGS at scale.^54^ Sustainability (what keeps the pathway alive after launch): Hybrid diagnostics fail most commonly during the “maintenance phase” when cartridges, controls, culture media, or staffing continuity break down. Programmes should therefore define a minimum sustainability package at launch: i) Protected time for at least one laboratory and one AMS lead to own SOPs and audit, ii) A small buffer stock policy for critical consumables and controls, iii) Basic downtime procedures (manual phone-out, paper reflex triggers) for power/LIS interruptions, and iv) Simple retention levers such as cross-training, clear role recognition (e.g., “RDT steward”), and documented escalation support from a tertiary/reference mentor site. These measures do not require high capital investment but materially reduce attrition of performance after the initial implementation period.

For metagenomic sequencing, multiple contemporary reviews converge on a similar set of translational challenges: high per sample cost, lack of standardised wet lab and bioinformatic pipelines, variable analytical sensitivity across specimen types, and difficulty distinguishing clinically meaningful reads from background contamination or commensal flora.^55^^,^^56^ A 2025 Diagnostics review adds regulatory uncertainty, absence of harmonised reporting standards and unresolved reimbursement models especially in resource constrained settings to this list.^57^ Ethical and data governance concerns about human genomic information generated alongside pathogen reads also complicate routine deployment.^58^

Beyond analytical constraints, two practical levers can improve the actionability of advanced diagnostics in LMIC hospitals: i) Triage-driven prioritisation of high-cost testing using simple sepsis screening approaches built from routinely available parameters (e.g., lymphocyte count, INR and procalcitonin), and ii) Explicit linkage of empiric/de-escalation pathways to population-specific epidemiology for example, a global synthesis in hematological malignancy cohorts summarizes the burden of *E. coli* bloodstream infection and associated resistance patterns that should be reflected in local guidance.^59^^,^^60^ In parallel, outcome studies in carbapenem-resistant Gram-negative infections show clinically meaningful differences between active regimens (e.g., ceftazidime-avibactam-based therapy versus polymyxin-based approaches), reinforcing the importance of timely optimisation of effective treatment.^61^^,^^62^

At the health system level, hybrid algorithms demand tight integration of diagnostic and antimicrobial stewardship. International frameworks now describe diagnostic stewardship as a formal continuum of AMS, requiring governance structures, agreed test ordering rules, and shared metrics rather than ad hoc local efforts. However, comparative effectiveness and cost utility data for different implementation models (centralised versus decentralised testing, hub-and-spoke sequencing services, or restricted indication order sets) remain limited, particularly in LMICs.

Pragmatic delivery model for sequencing (recommended for many LMIC systems): A hub-and-spoke approach can make Tier 3 achievable without forcing every hospital to insource sequencing. Spoke sites (district/secondary hospitals) define strict reflex criteria, collect and store specimens using a simple pre-analytical checklist, and ensure that Tier 2 culture/AST continues in parallel. The hub (tertiary/reference laboratory) runs validated wet-lab and bioinformatic pipelines, tracks contamination/QC metrics, and returns a report that is explicitly “action-oriented” (confidence level, likely pathogen versus background, and suggested confirmatory steps). Crucially, the service agreement should define realistic turnaround targets and an escalation pathway for time-critical ICU cases; otherwise, delayed sequencing risks becoming academically interesting but clinically irrelevant.

Key research gaps include: robust cost effectiveness studies of tiered algorithms in diverse settings prospective trials of diagnostic stewardship interventions specifically tailored to multiplex panels and mNGS development of simplified, quality assured mNGS workflows suitable for regional reference laboratories and models for workforce training and retention in microbiology and bioinformatics. Addressing these gaps will determine whether the proposed hybrid strategies can move from tertiary academic exemplars to scalable, equitable tools for global infectious disease care.

Practical note: “Accountable Role” in Table 1 is intentionally framed as a small cross-functional loop (laboratory + AMS + IPC), so that sites can implement the pathway through role-mapping and task-sharing rather than relying on a single specialist individual.

**Table 1:** Implementation and stewardship playbook for LMIC clinical microbiology laboratories.

Tier/Scenario	Operational Trigger	Mandated Clinical/Microbiology Action	Service Level / Turnaround Time	Accountable Role	Anticipated KPI Effect
Tier1: Bloodstream Infection Panel (BCID2)^16^	Reported pathogen with resistance determinant (e.g., mecA/C, CTX-M)	Initiate targeted therapy or de-escalate per local antibiogram: document rationale in the EMR	≤6h post-verification	On-duty microbiologist with AMS liaison	Reduced time-to-targeted therapy: higher de-escalation within 24 h
Tier 1: Lower Respiratory Tract Panel (PN/PNplus)^30^	Panel positive with low semi-quant bin and low pre-test probability	Avoid empiric broad-spectrum initiation: narrow/withhold pending corroboration: schedule 24 h re-evaluation	Immediate: structured re-review at 24h	Primary clinical team with AMS	Reduction in unnecessary broad-spectrum DOT
Tier 1: Any Critical Panel Result^53^	Result verified as critical (organism or marker)	Mandatory phone notification to clinical team and AMS: auto-insert interpretive comment regarding low-bin management	≤60 min from verification: LIS documentation required	On-duty microbiologist	Improved critical-result acknowledgment and action
Tier 2: Reflex Culture (BSI/LRTI)^30^	Organism class known phenotype pending	Rapid subculture: AST release ≤24h from positivity: discontinue duplicative coverage	≤24h after instrument flag	Culture bench lead	Shorter time-to-targeted therapy
Tier 2: Panel Culture Discordance^30^	Discordant or polymicrobial calls	Adjudicate dominance using semi quantitative data and phenotype streamline to a single targeted regimen	≤24‒48 h	Supervising microbiologist with AMS	Increased de-escalation within 24 h of definitive ID/AST
Tier 3: Sequencing (tNGS → mNGS)^40^^,^^55^^,^^58^	48‒72h culture negative, high clinical suspicion (e.g., CNS infection, severe sepsis)	Selective tNGS: escalate to mNGS if unresolved/high stakes: apply negative report stop rules	Order at 48‒72h: act on report same day	ID/AMS lead with send out coordinator	Higher management change rate with controlled utilization (target 5%‒15%)
Infection Prevention Escalation^53^	High risk pathogen identified	Initiate isolation order set notify IPC	Decision ≤4 h	IPC nurse lead	Timely isolation decisions reduced transmission risk
Governance and Monthly Audit^53^	Close of audit cycle	Export KPI dashboard if off-target across ≥2 cycles, initiate CAPA	Monthly	QA co-lead with AMS	Sustained KPI convergence
Contextual Anchor: Severe CAP Pathway	Panel plus AMS pathway implementation	Apply pathway per SOP for ICU/severe CAP	As per SOP	AMS with ICU/Pulmonology	Faster therapeutic adjustments reduced inappropriate use

**Fig. 1:**
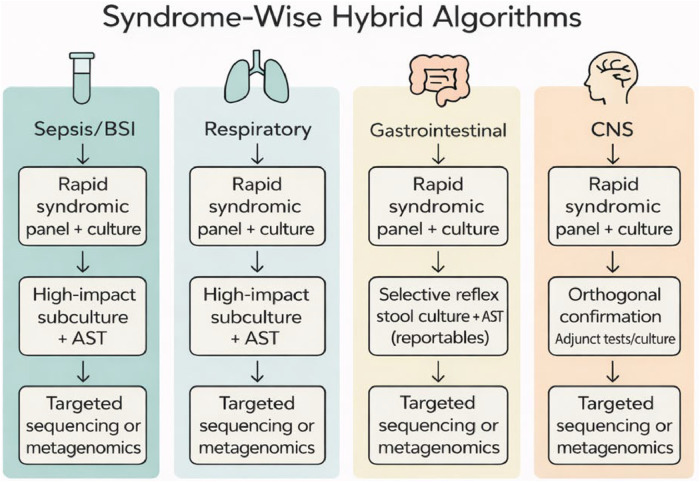
Syndrome-wise three-tier hybrid diagnostic algorithms.

Table 1 operationalises Fig. 1 by mapping each tier and trigger to mandated actions, service-level targets and accountable roles (lab + AMS + IPC).

## Limitations & future directions

Current evidence supporting hybrid tiered algorithms is dominated by single-centre studies and high-income tertiary hospitals, many of which already have reliable culture/AST, continuous staffing, and mature stewardship infrastructure. Data from low- and middle-income countries remain limited, so generalisability to laboratories facing personnel constraints, intermittent supplies, and minimal IT support is uncertain. The impact literature is also largely quasi-experimental, with heterogeneous endpoints and outcomes that are strongly mediated by stewardship intensity and co-interventions introduced during roll-out, this makes it difficult to isolate the independent contribution of any single modality (panel versus workflow versus AMS response). LMIC-specific cost-effectiveness analyses are sparse, particularly for sequencing, where per-sample cost, quality controls, bioinformatics, governance, and regulatory requirements can be prohibitive outside reference-laboratory models. Future work should prioritise pragmatic multicentre evaluations in LMIC health systems, compare delivery models (in-house versus hub-and-spoke), and report a consistent outcome set (time to effective therapy, broad-spectrum DOT, LOS, and cost per actionable diagnosis) alongside implementation measures (workforce time, supply resilience, and quality indicators).

## Conclusion

This review argues that modern microbiology is most effective when organised as a disciplined, three-tier hybrid pathway rather than as a loose collection of tests. At Tier 1, syndrome-specific rapid panels for sepsis, pneumonia, gastrointestinal infection, and CNS disease buy time: they move the team quickly from “any bug” to a short list of plausible pathogens and resistance markers, but they are deliberately not treated as stand-alone answers. Tier 2 reflex culture with targeted adjunct tests remains the non-negotiable specificity anchor, confirming viability, distinguishing infection from contamination or colonisation, providing phenotypic susceptibility data and supplying isolates for infection prevention and public health. Tier 3, targeted or metagenomic sequencing, is reserved for the small but clinically critical subset of high suspicion, culture negative cases where additional breadth can genuinely change management. Across all four syndromes, the message is the same: benefit comes not from technology alone but from how it is embedded. Clear indications, explicit reflex rules, tight linkage to antimicrobial stewardship and routine audit of key performance indicators are what convert panels, culture and sequencing into a coherent, accountable diagnostic service that can be adapted to both resource rich and resource limited settings.

## AI tools declaration

The authors used AI language models (such as ChatGPT, OpenAI) only to refine language and improve clarity. All content has been reviewed and approved by the authors, who take full responsibility for the final manuscript.

## Funding

None.

## Data availability statement

The data that support the findings of this study are available from the corresponding author upon reasonable request.

## Conflicts of interest

The authors declare no conflicts of interest.

## References

[1] Kozlakidis Z., Vandenberg O., Stelling J. (2020). Clinical microbiology in low resource settings. Front Med (Lausanne).

[2] Vineeth V.K., Nambi P.S., Gopalakrishnan R. (2024). Clinical utility of blood culture identification 2 panel in flagged blood culture samples from the intensive care unit of a tertiary care hospital. Indian J Crit Care Med.

[3] Salimnia H., Fairfax M.R., Lephart P.R. (2016). Evaluation of the FilmArray blood culture identification panel: results of a multicenter controlled trial. J Clin Microbiol.

[4] Alby K., Mitchell S.L. (2018). Lower respiratory multiplex panels for the detection of bacterial and viral infections. Clin Microbiol Newsl.

[5] He B., Zhu R., Yang H. (2020). Assessing the impact of data preprocessing on analyzing next generation sequencing data. Front Bioeng Biotechnol.

[6] Bou G., Cantón R., MartínezMartínez L., Navarro D., Vila J. (2021). Fundamentos e implementaciónde Programas de Optimización de Diagnóstico Microbiológico. Enferm Infecc Microbiol Clin.

[7] Senok A., Dabal L.A., Alfaresi M. (2023). Clinical impact of the BIOFIRE blood culture identification 2 panel in adult patients with bloodstream infection: a multicentre observational study in the United Arab Emirates. Diagnostics (Basel).

[8] Turner P., Ashley E.A., Celhay O.J. (2020). ACORN (A Clinically-Oriented Antimicrobial Resistance Surveillance Network): a pilot protocol for case-based antimicrobial resistance surveillance. Wellcome Open Res.

[9] Brendish N.J., Malachira A.K., Armstrong L. (2017). Routine molecular point-of-care testing for respiratory viruses in adults presenting to hospital with acute respiratory illness (ResPOC): a pragmatic, openlabel, randomised controlled trial. Lancet Respir Med.

[10] Srinivas P., Rivard K.R., Pallotta A.M. (2019). Implementation of a stewardship initiative on respiratory viral PCRbased antibiotic de-escalation. Pharmacotherapy.

[11] Webber D.M., Wallace M.A., Burnham C.A., Anderson N.W. (2020). Evaluation of the BioFire FilmArray pneumonia panel for detection of viral and bacterial pathogens in lower respiratory tract specimens in the setting of a tertiary care academic medical center. J Clin Microbiol.

[12] Zimmermann S., Horner S., Altwegg M., Dalpke A.H. (2020). Workflow optimization for syndromic diarrhea diagnosis using the molecular Seegene Allplex GIBacteria (I) assay. Eur J Clin Microbiol Infect Dis.

[13] Beal S.G., Tremblay E.E., Toffel S., Velez L., Rand K.H. (2018). A gastrointestinal PCR panel improves clinical management and lowers health care costs. J Clin Microbiol.

[14] Bhaskaran P.N., Moni M., Sathyapalan D.T. (2025). Impact of multiplex PCR respiratory viral panel testing on antibiotic utilisation in children with acute febrile and respiratory illnesses. BMJ Paediatr Open.

[15] Zakhour J., Haddad S.F., Kerbage A. (2023). Diagnostic stewardship in infectious diseases: a continuum of antimicrobial stewardship in the fight against antimicrobial resistance. Int J Antimicrob Agents.

[16] Rhoads D.D., Pournaras S., Leber A. (2023). Multicenter evaluation of the BIOFIRE blood culture identification 2 panel for detection of bacteria, yeasts, and antimicrobial resistance genes in positive blood culture samples. J Clin Microbiol.

[17] Olvera A., Carter H., Rajan A. (2021). Enteropathogenic *Escherichia coli* infection in cancer and immunosuppressed patients. Clin Infect Dis.

[18] Reller L.B., Weinstein M.P., Murdoch D.R (2003). Nucleic acid amplification tests for the diagnosis of pneumonia. Clin Infect Dis.

[19] Hanson K.E., Couturier M.R. (2016). Multiplexed molecular diagnostics for respiratory, gastrointestinal, and central nervous system infections. Clin Infect Dis.

[20] Ling D., Seidelman J., Dodds-Ashley E. (2020). Navigating reflex urine culture practices in community hospitals: need for a validated approach. Am J Infect Control.

[21] Sibley C.D., Peirano G., Church D.L. (2012). Molecular methods for pathogen and microbial community detection and characterization: current and potential application in diagnostic microbiology. Infect Genet Evol.

[22] Afshari A., Schrenzel J., Ieven M., Harbarth S. (2012). Bench-to-bedside review: rapid molecular diagnostics for bloodstream infection ‒ a new frontier?. Crit Care.

[23] Smith K.P., Kirby J.E. (2019). Rapid susceptibility testing methods. Clin Lab Med.

[24] Morency-Potvin P., Schwartz D.N., Weinstein R.A. (2017). Antimicrobial stewardship: how the microbiology laboratory can right the ship. Clin Microbiol Rev.

[25] Humphries R.M., Dien Bard J. (2016). Point-counterpoint: reflex cultures reduce laboratory workload and improve antimicrobial stewardship in patients suspected of having urinary tract infections. J Clin Microbiol.

[26] Park S.Y., Chang E.J., Ledeboer N. (2023). Plasma microbial cell-free DNA sequencing from over 15,000 patients identified a broad spectrum of pathogens. J Clin Microbiol.

[27] Chiu C.Y., Miller S.A. (2019). Clinical metagenomics. Nat Rev Genet.

[28] Schlaberg R., Chiu C.Y., Miller S., Procop G.W., Weinstock G. (2017). Validation of metagenomic nextgeneration sequencing tests for universal pathogen detection. Arch Pathol Lab Med.

[29] Gescher D.M., Kovacevic D., Schmiedel D. (2008). Fluorescence in situ hybridisation (FISH) accelerates identification of Gram-positive cocci in positive blood cultures. Int J Antimicrob Agents.

[30] Murphy C.N., Fowler R., BaladaLlasat J.M. (2020). Multicenter evaluation of the BioFire FilmArray pneumonia/pneumonia plus panel for detection and quantification of agents of lower respiratory tract infection. J Clin Microbiol.

[31] Monard C., Pehlivan J., Auger G. (2020). Multicenter evaluation of a syndromic rapid multiplex PCR test for early adaptation of antimicrobial therapy in adult patients with pneumonia. Crit Care.

[32] Buchan B.W., Windham S., BaladaLlasat J.M. (2020). Practical comparison of the BioFire FilmArray pneumonia panel to routine diagnostic methods and potential impact on antimicrobial stewardship in adult hospitalised patients with lower respiratory tract infections. J Clin Microbiol.

[33] Van Der Westhuyzen M., Samodien N., Brink A.J., Moodley C. (2023). Utility of the BioFire FilmArray Pneumonia Panel plus assay for syndromic testing of lower respiratory tract infections in a low/middleincome setting. JAC Antimicrob Resist.

[34] Stratton C.W., Schutzbank T.E., Tang Y.W. (2021). Use of metagenomic next-generation sequencing in the clinical microbiology laboratory: a step forward, but not an end-all. J Mol Diagn.

[35] Saif N.T., Dooley C., Baghdadi J.D., Morgan D.J., Coffey K.C. (2024). Clinical decision support for gastrointestinal panel testing. Antimicrob Steward Healthc Epidemiol.

[36] Kanwar N., Jackson J., Bardsley T. (2023). Impact of rapid molecular multiplex gastrointestinal pathogen testing in management of children during a Shigella outbreak. J Clin Microbiol.

[37] Decuir M., Fowler R.C., Cebelinski E., Smith K., Boxrud D., Medus C. (2021). Evidence of false positivity for Vibrio species tested by gastrointestinal multiplex PCR panels, Minnesota, 2016–2018. Open Forum Infect Dis.

[38] Buss S.N., Leber A., Chapin K. (2015). Multicenter evaluation of the BioFire FilmArray gastrointestinal panel for etiologic diagnosis of infectious gastroenteritis. J Clin Microbiol.

[39] TrujilloGomez J., Tsokani S., ArangoFerreira C. (2022). BioFire FilmArray Meningitis/Encephalitis panel for the aetiological diagnosis of central nervous system infections: a systematic review and diagnostic test accuracy meta-analysis. EClinicalMedicine.

[40] Benoit P., Brazer N., de Lorenzi-Tognon M. (2024). Sevenyear performance of a clinical metagenomic nextgeneration sequencing test for diagnosis of central nervous system infections. Nat Med.

[41] Zhu Y., Gan M., Ge M. (2023). Diagnostic performance and clinical impact of metagenomic nextgeneration sequencing for pediatric infectious diseases. J Clin Microbiol.

[42] Zhou L., Zou X., Yong Y., Hu Q. (2024). Using cerebrospinal fluid nanopore sequencing assay to diagnose tuberculous meningitis: a retrospective cohort study in China. BMJ Open.

[43] Peri A.M., Chatfield M.D., Ling W., Furuya-Kanamori L., Harris P.N., Paterson D.L. (2024). Rapid diagnostic tests and antimicrobial stewardship programs for the management of bloodstream infection: what is their relative contribution to improving clinical outcomes? A systematic review and network metaanalysis. Clin Infect Dis.

[44] Chen H.Y., Tseng H.Y., Chen C.L. (2024). The real-world impact of the BioFire FilmArray Blood Culture Identification 2 Panel on antimicrobial stewardship among patients with bloodstream infections in intensive care units with a high burden of drugresistant pathogens. J Microbiol Immunol Infect.

[45] Baum A., Miller J.L., Gavaghan V., Beck E.T., Argotsinger J. (2025). Effect of biofire blood culture identification 2 (BCID2) panel versus a matrix-assisted laser desorption/ionization-time of flight mass spectrometry (MALDITOF MS) rapid incubation protocol on time to optimal therapy in patients with positive blood cultures. BMC Infect Dis.

[46] Huang B.M., Lo C.L., Lin W.L. (2026). Application of antimicrobial stewardship interventions improves outcomes in adults with bloodstream infection caused by multidrug-resistant Enterobacteriaceae. J Microbiol Immunol Infect.

[47] Virk A., Strasburg A.P., Kies K.D. (2024). Rapid multiplex PCR panel for pneumonia in hospitalised patients with suspected pneumonia in the USA: a single-centre, open-label, pragmatic, randomised controlled trial. Lancet Microbe.

[48] Miller M.M., Van Schooneveld T.C., Stohs E.J., Marcelin J.R., Alexander B.T., Watkins A.B. (2023). Implementation of a rapid multiplex polymerase chain reaction pneumonia panel and subsequent antibiotic deescalation. Open Forum Infect Dis.

[49] Ilges D., Graf E.H., Grant L. (2025). Positive impact of a diagnostic stewardship intervention on syndromic panel ordering practices and inappropriate Clostridioides difficile treatment. Infect Control Hosp Epidemiol.

[50] Xu F., Chen C., Lu S., Xue M., Ding H., Song Y. (2025). Impact of metagenomics nextgeneration sequencing on etiological diagnosis and early outcomes in sepsis. J Transl Med.

[51] Lai L.M., Dai Q.B., Cao M.L., Liu Y., Zhao R., Yuan L. (2025). Clinical utility of metagenomic nextgeneration sequencing in pathogen detection for lower respiratory tract infections. Sci Rep.

[52] Simons C.C., Capraro G.A. (2024). Barriers to implementation of rapid identification and antimicrobial susceptibility testing technologies in the clinical microbiology laboratory: an American perspective. J Antimicrob Chemother.

[53] Dumm R.E., Marlowe E.M., Patterson L., Larkin P.M., She R.C., Filkins L.M. (2024). The foundation for the microbiology laboratory’s essential role in diagnostic stewardship: an ASM laboratory practices subcommittee report. J Clin Microbiol.

[54] Pauwels I., Versporten A., Ashiru-Oredope D., Costa S.F., Maldonado H., Porto A.P. (2025). Implementation of hospital antimicrobial stewardship programmes in low- and middleincome countries: a qualitative study from a multi-professional perspective in the Global-PPS network. Antimicrob Resist Infect Control.

[55] Gaston D.C. (2023). Clinical metagenomics for infectious diseases: progress toward operational value. J Clin Microbiol.

[56] Batool M., GallowayPeña J. (2023). Clinical metagenomics ‒ challenges and future prospects. Front Microbiol.

[57] Elbehiry A., Abalkhail A. (2025). Metagenomic nextgeneration sequencing in infectious diseases: clinical applications, translational challenges, and future directions. Diagnostics (Basel).

[58] Rodino K.G., Simner P.J. (2024). Status check: nextgeneration sequencing for infectiousdisease diagnostics. J Clin Invest.

[59] Jafarabadi M., Khaledi A. (2024). *Escherichia coli* bloodstream infections and associated antibiotic resistance pattern in hematological malignancy populations: a global systematic review. Eurasian J Med Oncol.

[60] Liu B., Du H., Zhang J. (2022). Developing a new sepsis screening tool based on lymphocyte count, international normalized ratio and procalcitonin (LIP score). Sci Rep.

[61] Zhuang H.H., Qu Q., Long W.M. (2025). Ceftazidime/avibactam versus polymyxin B in carbapenem-resistant Klebsiella pneumoniae infections: a propensity score-matched multicenter real-world study. Infection.

[62] Zhuang H.H., Chen Y., Hu Q. (2023). Efficacy and mortality of ceftazidime/avibactam-based regimens in carbapenem-resistant Gram-negative bacteria infections: a retrospective multicenter observational study. J Infect Public Health.

